# Supplementary material for: Genetic affinity between Ningxia Hui and eastern Asian populations revealed by a set of InDel loci
Source: R Soc Open Sci. 2020 Jan 8;7(1):190358. doi: 10.1098/rsos.190358 (PMC7029925; doi:10.1098/rsos.190358)
Supplement: Tables S1 - S3 [file rsos190358supp1.docx]

| Supplementary Table 1. Pairwise *Fst* values between Ningxia Hui and other 21 populations. | | | | | | | | | | | | | | | | | | | | | | |
| --- | --- | --- | --- | --- | --- | --- | --- | --- | --- | --- | --- | --- | --- | --- | --- | --- | --- | --- | --- | --- | --- | --- |
| Populations | BeijingHan | GuangdongHan | ShanghaiHan | Yi | Xibe | Tibetan | South  Korean | She | Dane | Hungarian | Basque | CentralSpanish | Uruguayan | Kazak | Uigur | **Hui** | Chihuahua-Mexican | Jalisco-Mexican | Mexico-Mexican | Veracruz-Mexican | Yucatan-Mexican | Amerindian-Mexican |
| BeijingHan | 0.0000 |  |  |  |  |  |  |  |  |  |  |  |  |  |  |  |  |  |  |  |  |  |
| GuangdongHan | 0.0037 | 0.0000 |  |  |  |  |  |  |  |  |  |  |  |  |  |  |  |  |  |  |  |  |
| ShanghaiHan | 0.0014 | 0.0010 | 0.0000 |  |  |  |  |  |  |  |  |  |  |  |  |  |  |  |  |  |  |  |
| Yi | 0.0182 | 0.0124 | 0.0139 | 0.0000 |  |  |  |  |  |  |  |  |  |  |  |  |  |  |  |  |  |  |
| Xibe | 0.0050 | 0.0073 | 0.0045 | 0.0178 | 0.0000 |  |  |  |  |  |  |  |  |  |  |  |  |  |  |  |  |  |
| Tibetan | 0.0067 | 0.0175 | 0.0122 | 0.0218 | 0.0103 | 0.0000 |  |  |  |  |  |  |  |  |  |  |  |  |  |  |  |  |
| SouthKorean | 0.0059 | 0.0051 | 0.0019 | 0.0142 | 0.0038 | 0.0113 | 0.0000 |  |  |  |  |  |  |  |  |  |  |  |  |  |  |  |
| She | 0.0032 | 0.0033 | 0.0043 | 0.0173 | 0.0092 | 0.0187 | 0.0070 | 0.0000 |  |  |  |  |  |  |  |  |  |  |  |  |  |  |
| Dane | 0.0811 | 0.0916 | 0.0918 | 0.1047 | 0.0782 | 0.0703 | 0.0961 | 0.0932 | 0.0000 |  |  |  |  |  |  |  |  |  |  |  |  |  |
| Hungarian | 0.0801 | 0.0921 | 0.0923 | 0.1032 | 0.0779 | 0.0673 | 0.0958 | 0.0942 | 0.0060 | 0.0000 |  |  |  |  |  |  |  |  |  |  |  |  |
| Basque | 0.0885 | 0.0930 | 0.0937 | 0.1108 | 0.0818 | 0.0818 | 0.0961 | 0.0996 | 0.0119 | 0.0124 | 0.0000 |  |  |  |  |  |  |  |  |  |  |  |
| CentralSpanish | 0.0843 | 0.0919 | 0.0921 | 0.1066 | 0.0769 | 0.0713 | 0.0952 | 0.0963 | 0.0054 | 0.0040 | 0.0053 | 0.0000 |  |  |  |  |  |  |  |  |  |  |
| Uruguayan | 0.0724 | 0.0825 | 0.0818 | 0.0923 | 0.0683 | 0.0601 | 0.0839 | 0.0843 | 0.0108 | 0.0050 | 0.0108 | 0.0036 | 0.0000 |  |  |  |  |  |  |  |  |  |
| Kazak | 0.0219 | 0.0307 | 0.0296 | 0.0415 | 0.0194 | 0.0168 | 0.0338 | 0.0350 | 0.0309 | 0.0284 | 0.0368 | 0.0270 | 0.0214 | 0.0000 |  |  |  |  |  |  |  |  |
| Uigur | 0.0274 | 0.0367 | 0.0354 | 0.0517 | 0.0281 | 0.0234 | 0.0404 | 0.0417 | 0.0272 | 0.0226 | 0.0311 | 0.0209 | 0.0178 | -0.0001 | 0.0000 |  |  |  |  |  |  |  |
| **Hui** | **0.0014** | **0.0033** | **0.0008** | **0.0158** | **0.0011** | **0.0073** | **0.0025** | **0.0047** | **0.0672** | **0.0677** | **0.0729** | **0.0685** | **0.0599** | **0.0166** | **0.0220** | **0.0000** |  |  |  |  |  |  |
| Chihuahua-Mexican | 0.1487 | 0.1621 | 0.1620 | 0.1731 | 0.1533 | 0.1402 | 0.1702 | 0.1758 | 0.0635 | 0.0544 | 0.0733 | 0.0687 | 0.0724 | 0.0972 | 0.0844 | **0.1429** | 0.0000 |  |  |  |  |  |
| Jalisco-Mexican | 0.1456 | 0.1551 | 0.1557 | 0.1711 | 0.1462 | 0.1373 | 0.1647 | 0.1700 | 0.0569 | 0.0460 | 0.0661 | 0.0602 | 0.0645 | 0.0895 | 0.0773 | **0.1361** | -0.0011 | 0.0000 |  |  |  |  |
| Mexico-Mexican | 0.1755 | 0.1845 | 0.1841 | 0.1981 | 0.1753 | 0.1638 | 0.1916 | 0.2001 | 0.0740 | 0.0696 | 0.0863 | 0.0861 | 0.0918 | 0.1170 | 0.1059 | **0.1643** | 0.0117 | 0.0062 | 0.0000 |  |  |  |
| Veracruz-Mexican | 0.1651 | 0.1713 | 0.1727 | 0.1880 | 0.1631 | 0.1534 | 0.1804 | 0.1866 | 0.0648 | 0.0538 | 0.0704 | 0.0659 | 0.0760 | 0.1035 | 0.0902 | **0.1518** | 0.0089 | -0.0003 | 0.0034 | 0.0000 |  |  |
| Yucatan-Mexican | 0.1920 | 0.1997 | 0.2000 | 0.2183 | 0.1911 | 0.1835 | 0.2078 | 0.2146 | 0.0904 | 0.0774 | 0.0944 | 0.0940 | 0.1013 | 0.1328 | 0.1194 | **0.1817** | 0.0121 | 0.0091 | 0.0083 | 0.0041 | 0.0000 |  |
| Amerindian-Mexican | 0.2258 | 0.2291 | 0.2307 | 0.2483 | 0.2225 | 0.2175 | 0.2399 | 0.2494 | 0.1176 | 0.0988 | 0.1275 | 0.1262 | 0.1347 | 0.1668 | 0.1530 | **0.2139** | 0.0248 | 0.0208 | 0.0234 | 0.0138 | 0.0129 | 0.0000 |

| Supplementary Table 2. Results of the three population test *f*_3_ (*C*;*A*,*B*) *C*: Hui, Uigur or Kazak; *A*: Dane or CentralSpanish; *B*: three Han populations | | | | | |
| --- | --- | --- | --- | --- | --- |
|  |  |  |  |  |  |
| A | B | C | *f*_3_ | std.err | Z-score |
| Dane | BeijingHan | hui | -0.007611 | 0.004146 | -1.836 |
| Dane | GuangdongHan | hui | -0.01035 | 0.00354 | -2.924 |
| Dane | ShanghaiHan | hui | -0.011132 | 0.003424 | -3.251 |
| Dane | BeijingHan | Uigur | -0.014142 | 0.005988 | -2.362 |
| Dane | GuangdongHan | Uigur | -0.013766 | 0.005546 | -2.482 |
| Dane | ShanghaiHan | Uigur | -0.014193 | 0.005932 | -2.393 |
| Dane | BeijingHan | Kazak | -0.015322 | 0.006577 | -2.33 |
| Dane | GuangdongHan | Kazak | -0.015028 | 0.006277 | -2.394 |
| Dane | ShanghaiHan | Kazak | -0.015295 | 0.006678 | -2.29 |
| CentralSpanish | BeijingHan | hui | -0.008728 | 0.004115 | -2.121 |
| CentralSpanish | GuangdongHan | hui | -0.009695 | 0.003901 | -2.485 |
| CentralSpanish | ShanghaiHan | hui | -0.010558 | 0.003706 | -2.849 |
| CentralSpanish | BeijingHan | Uigur | -0.019036 | 0.006073 | -3.135 |
| CentralSpanish | GuangdongHan | Uigur | -0.017068 | 0.005657 | -3.017 |
| CentralSpanish | ShanghaiHan | Uigur | -0.017569 | 0.006019 | -2.919 |
| CentralSpanish | BeijingHan | Kazak | -0.019051 | 0.006822 | -2.793 |
| CentralSpanish | GuangdongHan | Kazak | -0.017139 | 0.006578 | -2.606 |
| CentralSpanish | ShanghaiHan | Kazak | -0.01748 | 0.006929 | -2.523 |

| Supplementary Table 3. The three population test in the outgroup case *f*_3_ (*C*;*A*,*B*) *C*:Chihuahua-Mexican or Dane; *A*: Hui; *B*: East Asian populations | | | | | |
| --- | --- | --- | --- | --- | --- |
|  |  |  |  |  |  |
| A | B | C | f3 | std.err | Z-score |
| hui | Uigur | Chihuahua-Mexican | 0.115053 | 0.023382 | 4.921 |
| hui | Kazak | Chihuahua-Mexican | 0.12524 | 0.024092 | 5.198 |
| hui | Tibetan | Chihuahua-Mexican | 0.153785 | 0.03014 | 5.102 |
| hui | BeijingHan | Chihuahua-Mexican | 0.160974 | 0.03141 | 5.125 |
| hui | Xibe | Chihuahua-Mexican | 0.162667 | 0.029168 | 5.577 |
| hui | GuangdongHan | Chihuahua-Mexican | 0.16511 | 0.031728 | 5.204 |
| hui | ShanghaiHan | Chihuahua-Mexican | 0.165665 | 0.031311 | 5.291 |
| hui | Yi | Chihuahua-Mexican | 0.166986 | 0.032177 | 5.19 |
| hui | SouthKorean | Chihuahua-Mexican | 0.169717 | 0.031783 | 5.34 |
| hui | She | Chihuahua-Mexican | 0.174824 | 0.032909 | 5.312 |
| hui | Uigur | Dane | 0.036786 | 0.010108 | 3.639 |
| hui | Kazak | Dane | 0.041294 | 0.010483 | 3.939 |
| hui | Tibetan | Dane | 0.065433 | 0.019497 | 3.356 |
| hui | BeijingHan | Dane | 0.073388 | 0.019396 | 3.784 |
| hui | Xibe | Dane | 0.070879 | 0.018513 | 3.829 |
| hui | GuangdongHan | Dane | 0.075803 | 0.018743 | 4.044 |
| hui | ShanghaiHan | Dane | 0.076508 | 0.019465 | 3.931 |
| hui | Yi | Dane | 0.078138 | 0.019432 | 4.021 |
| hui | SouthKorean | Dane | 0.077995 | 0.020427 | 3.818 |
| hui | She | Dane | 0.077172 | 0.017619 | 4.38 |
